# Identification of genes differentially expressed in dorsal and ventral chick midbrain during early Development

**DOI:** 10.1186/1471-213X-9-29

**Published:** 2009-04-27

**Authors:** A Chittka, JN Volff, A Wizenmann

**Affiliations:** 1 Junior Research Group, Biozentrum, Am Hubland, 97074, Würzburg, Germany; 2 Institut de Génomique Fonctionnelle de Lyon; Université de Lyon; Institut Fédératif Biosciences Gerland Lyon Sud; Université Lyon 1, CNRS, INRA, Ecole Normale Supérieure de Lyon, France; 3 MRC Laboratory for Molecular Cell Biology, UCL, Gower Street, London, WC1E 6BT, UK; 4 Current address: Institute for Anatomy, Experimental Embryology, Österbergstrasse, Tübingen, Germany

## Abstract

**Background:**

During the development of the central nervous system (CNS), patterning processes along the dorsoventral (DV) axis of the neural tube generate different neuronal subtypes. As development progresses these neurons are arranged into functional units with varying cytoarchitecture, such as laminae or nuclei for efficient relaying of information. Early in development ventral and dorsal regions are similar in size and structure. Different proliferation rates and cell migration patterns are likely to result in the formation of laminae or nuclei, eventually. However, the underlying molecular mechanisms that establish these different structural arrangements are not well understood.

We undertook a differential display polymerase chain reaction (DD-PCR) screen to identify genes with distinct expression patterns between dorsal and ventral regions of the chick midbrain in order to identify genes which regulate the sculpturing of such divergent neuronal organisation. We focused on the DV axis of the early chick midbrain since mesencephalic alar plate and basal plate develop into laminae and nuclei, respectively.

**Results:**

We identified 53 differentially expressed bands in our initial screen. Twenty-six of these could be assigned to specific genes and we could unambiguously show the differential expression of five of the isolated cDNAs in vivo by *in situ *mRNA expression analysis. Additionally, we verified differential levels of expression of a selected number of genes by using reverse transcriptase (RT) PCR method with gene-specific primers.

One of these genes, *QR1*, has been previously cloned and we present here a detailed study of its early developmental time course and pattern of expression providing some insights into its possible function. Our phylogenetic analysis of *QR1 *shows that it is the chick orthologue of *Sparc-like 1/Hevin/Mast9 *gene in mice, rats, dogs and humans, a protein involved in cell adhesion.

**Conclusion:**

This study reveals some possible networks, which might be involved in directing the difference in neuronal specification and cytoarchitecture observed in the brain.

## Background

The precise neural networks of the adult brain are a consequence of early embryonic development, when nerve cells acquire their unique identity, location and connections. Early in development the neural tube is regionalized along its anterior-posterior (AP) axis into forebrain, midbrain, hindbrain and spinal cord [1]. Shortly after, the dorso-ventral (DV) axis is determined [2-6]. This morphological development is accompanied by the expression of specific transcription factors, which dictate the overall plan of the central nervous system (CNS). Within each region a large diversity of neurons appears in a precise spatial and temporal pattern [3].

Brain regions not only differ in their neuronal subtypes, but also in their cytoarchitectural structures – they can either form laminae or nuclei. Such functional organization within the brain requires that the newborn neurons not only acquire the correct phenotype, but that they migrate to the appropriate positions within the brain. Multiple factors such as different types of neuronal migration, cell division patterns and the identity of specific neurons, contribute to the final cytoarchitectural organization of the CNS. For example, radial migration of neurons prevails in the generation of laminae, whereas nuclei are formed as a result of more tangential migration. Furthermore, differences in the pattern of cell division have been suggested to influence the formation of laminae and nuclei. An initial higher symmetric division of progenitor cells in the ventricular zone results in a more lateral extension of the neuroepithelium, a feature observed in many laminae forming brain regions [7]. There is also increasing evidence that early events, which specify neuronal identity, also influence the migratory patterns these neurons will follow. The proneural basic helix-loop-helix (bHLH) transcription factors Neurogenin 1 and 2 (Ngn1 and Ngn2) not only specify neuronal versus glial fates, but also support neuronal migration [8-10]. Thus, the processes of neurogenesis and fate specification may be intimately co-regulated with the subsequent migration and final connectivity from a very early stage of neuronal development.

Several genes have been implicated in forming the right laminar structure in the mammalian cortex. One of the best studied pathways of altered neuronal migration is exemplified by the *reelin *and *mdab *genes, whose mutations affect neuronal migration and laminar architecture of the cerebral cortex, hippocampus and cerebellum [11]. The superficial layers of the superior colliculus of the reelin-deficient mice show some defects; however, they are not as pronounced as in the other areas of the brain [12]. Another complex of proteins that affects neuronal migration is the microtubule motor complex, which includes dynein, dynactin, Lis1 and Nde1. Loss of function of these proteins leads to defects in cortical lamination [13-16]. Interestingly, mice which lack Lis1 gene also exhibit a reduction in neuroblast proliferation [17] and mice lacking Nde1 are characterized by a small brain [18]. Taken together, these results emphasize the importance of coordinated regulation of neuronal precursor proliferation, fate specification and migration (for reviews see [19,20]).

The aim of this study was to identify known or novel candidate genes with potential roles in the regulation of differential cytoarchitecture and neuronal specificity observed in the midbrain. The chick midbrain is one of the brain regions, which gives rise to laminae and nuclei. Dorsally, in the tectum, neurons are organized in layers and receive and process visual and auditory information. Ventrally, the tegmentum forms discrete nuclei including the substantia nigra where the dopaminergic neurons affected in Parkinson's disease are found. Early in development ventral and dorsal parts of the mesencephalon are similar in size and structure. After embryonic day 3 of chick development the dorsal half of the midbrain undergoes rapid cell proliferation to form an enlarged tectum. This massive enlargement along the medio-lateral axis is not observed in the ventral midbrain, where immigrating cells from the rhombic lip contribute, for example, to the isthmic nuclei in birds ([21] and references therein). Thus, the midbrain provides an ideal model system to address the question of molecular mechanisms orchestrating differential neuronal cytoarchitecture. We used a differential display (DD) screen [22,23] to identify genes with different expression patterns between ventral and dorsal midbrain of the chick some of which might be involved in regulating DV patterning of the mesencephalon.

Different methods have been designed to identify and sample the different sets of genes expressed by specific tissue or cell types. Although the preferred current method for screens is a microarray, we feel that there is less bias in the production of a representative library from the tissue directly rather than relying on the microarray representation. Compared to a microarray, it is a cheap method, which might identify novel genes that haven't been annotated yet. In addition, at the time this screen was initiated, chicken based microarray systems were not available. Taken together a differential display is ideally suited for addressing developmental questions. We used chick embryos between stages HH 9–11 [24] to isolate midbrains. Separate cDNA representation libraries were prepared from dorsal and ventral parts of the midbrains and compared. cDNA bands that showed differential representation were isolated to verify the expression patterns *in vivo *by *in situ *RNA hybridisation analysis of whole mount chick embryos at the appropriate stages of development and/or with RT-PCR.

In our initial screen we identified 53 bands, which were differentially represented between the dorsal and ventral parts of the midbrain. Twenty-six of these bands could be assigned to known genes and are listed in this work. Additional 8 bands could not be assigned to any known genes. Their chromosomal locations within the genome are presented. To date we do not have sequence information about the remaining 19 bands isolated in the screen. The differential expression pattern for five of the genes was verified by RT-PCR and by *in situ *mRNA hybridization to validate the screen. We also present a detailed expression study of one of the clones, M11d1, that was found to correspond to a previously identified gene, called *QR1 *[25,26].

## Results and discussion

### Differential Display of mRNAs from the dorsal and ventral Parts of the Chick Midbrain

**Figure 1:**
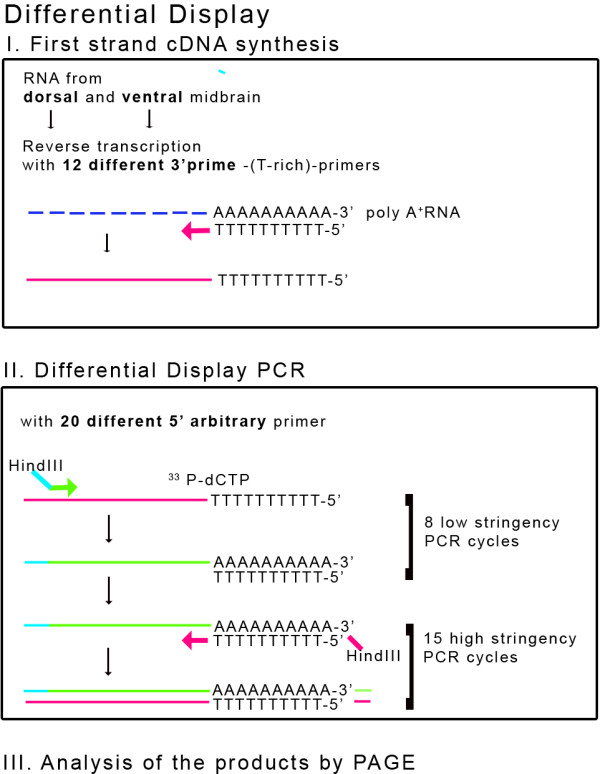
**Schematic representation of the differential display PCR screen**. An outline of the differential display-PCR technique utilised in the screen.

To identify genes with different spatial distribution between dorsal and ventral chick midbrain, we chose to use embryos from developmental stages HH 9–11 for our screen. At these stages there is no obvious anatomical difference between the ventral and dorsal parts of the midbrain, although the anterior-posterior subdivisions of the neural tube into the different parts of the brain are clearly visible. A schematic drawing of our screening strategy is presented in Fig. 1 and detailed in the materials and methods section. Here, we present the results of the 40 primer combinations we used in the initial screen.

**Figure 2:**
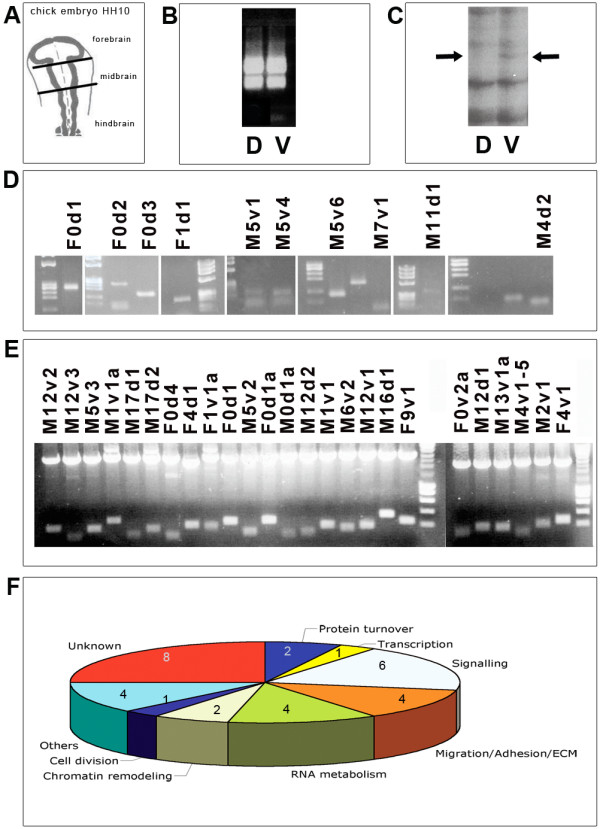
**Examples of intermediate steps of the differential display PCR screen**. (A). The two solid black lines in this schematic representation of a HH10 chick embryo indicate where the midbrains were excised. (B). Total RNA run on an ethidium bromide stained agarose gel displaying prominent 28S and 18S RNA bands. Total RNA was isolated from ventral and dorsal midbrains. (C). The gel shows the differentially expressed cDNA of the M5v4 clone after DD-PCR had been performed. (D). Examples of 10 PCR amplified products separated on a 2% agarose gels after their isolation from the sequencing gels and re-amplification. The primer combinations used are indicated above the gels. (E). Twenty five of the inserts obtained after subcloning the PCR products into pBS plasmid for subsequent sequencing. The primer combinations used are shown on the panel. (F). Functional classification of the genes isolated from this screen.

**Figure 3:**
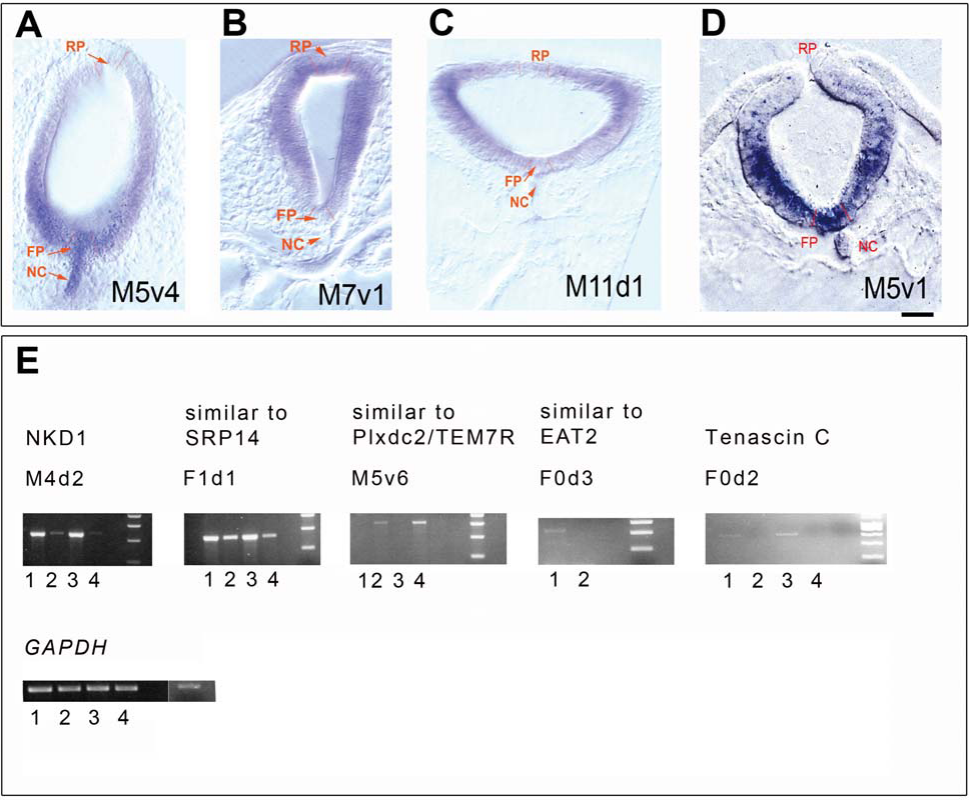
**Verification of the differential expression of the cDNAs excised from the gels by mRNA *in situ *hybridisation of chick midbrain**. (A-D) show the expression patterns of the mRNA coding for the transcripts isolated with the primer combinations indicated on the panels. Embryos were sectioned coronally, after whole mount *in situ *hybridisation at HH10. (E). Products of RT-PCR with gene-specific primers to verify differential expression of the transcripts in the dorsal and ventral parts of chick midbrains. Lanes are as follows: 1) HH 11 dorsal cDNA, 2) HH11 ventral cDNA, 3) HH16 dorsal cDNA, 4) HH16 ventral cDNA. The gene names and primer combinations are indicated above the relevant panels. GAPDH was used to normalize the cDNA expression levels. The equal amount of GAPDH amplified from ventral and dorsal midbrain cDNA demonstrates that there is no bias in the different cDNAs used.

The overview of the different steps of the screen is shown on figures 2A–D. Midbrains were excised as outlined in Fig. 2A and cut into dorsal and ventral parts, which were then processed for total RNA isolation. We performed three independent dissections and collections of embryos to isolate total mRNA, which was subsequently used for three independent cDNA syntheses. We assessed the integrity of the isolated RNA by agarose gel electrophoresis and staining the samples with ethidium bromide to visualize the 28S and 18S RNA (Fig. 2B). The cDNA products were amplified using radioactive nucleotides and different primer combinations by PCR amplification and separated on sequencing polyacrylamide gels. Representations from the ventral and dorsal cDNA were compared and bands, which showed differential representation, were excised from the gel (Fig. 2C displays an example of such a band, M5v4). All sample synthesis and amplification were performed in triplicate and only those bands, where all three PCR fragments from a particular primer combination showed differential representation were used for subsequent analysis. Bands isolated from sequencing gels were further amplified with the same primers that were used in the first round of amplification. We identified 53 differentially represented bands in our initial screen that were revealed by PCR analysis of the isolated cDNA. Of these, 19 were isolated from the ventral part of the midbrain and 34 from the dorsal part. The PCR products were separated on an agarose gel for further isolation and subcloning into a plasmid vector (Fig. 2D shows examples of some re-amplified cDNA products with different primer combinations and Fig. 2E shows the inserts retrieved after subcloning the amplified products into pBS plasmid). A schematic representation of the different functional subclasses of genes identified in the screen is presented in Fig. 2F. Employing RNA *in situ *hybridisation we not only validated a differential dorso-ventral expression of the candidate genes, but also detected the actual distribution of the mRNA transcripts in the chick midbrain at the appropriate stage (HH10). We used the original cDNA that was isolated and amplified from the acrylamide gels as templates for mRNA detection in *in situ *analysis. Antisense and sense RNA of the cDNA fragments were transcribed *in vitro *using T3 and T7 RNA polymerases. Of the clones, which were analysed in this manner we could unambiguously show that at least four (M5v1, M5v4, M7v1, and M11d1) had differential dorso-ventral expression pattern in the midbrain at HH10. The expression patterns of these four genes in the midbrain of the chick embryo at HH10 are shown on Fig. 3. M5v4 shows a clear dorso-ventral gradient with much stronger expression in the ventral region of the neural tube (Fig. 3A). M7v1 displays a dorso-ventral gradient with a stronger dorsal expression in the neural tube (Fig. 3B). M11d1 shows a rather interesting pattern of expression with the highest concentration at the medio-lateral part of the neural tube (Fig. 3C). M5v1 has a clear strong expression in the ventral aspect of the midbrain (Fig. 3D). We also verified different dorso-ventral expression levels by performing RT-PCR amplification with gene-specific primers for a selected group of genes to further validate the screening strategy (see Fig. 3E). The RT-PCR from cDNA of ventral and dorsal midbrain confirmed the original differential expression of the clones found in the DD-PCR. Clone M4d2 is strongly expressed in dorsal midbrain at HH10 and HH16, weakly in ventral midbrain at HH10 but has disappeared ventrally at HH16. F1d1 is present in ventral and dorsal midbrain at HH10 and HH16 but has a stronger expression in dorsal midbrain. F0d3 and F0d2 are only expressed in dorsal midbrain and M5v6 is only present in ventral midbrain. A summary of the gene identities obtained from this screen by comparing representational libraries from dorsal and ventral tissues is presented in table 1. Sequence information for the DNA fragments with no homology to any of the known genes is shown in table 2 along with the chromosomal locations of the sequences.

**Table 1:** Summary of cDNA fragments identified by the DD-PCR screen of ventral and dorsal midbrain regions.

**Clone ID**	**Gene Bank** **accession number**	**Gene product**	**Function**	**Expression**
M5v3	XM_001236352.1	Kinase (PRKA) anchor protein 8-like (AKAP8L)	Cell division	n.d.
M12v2	NM_001031296.1	Splicing factor, arginine/serine-rich 11 SFRS11	RNA processing	n.d.
F1d1	NW_001471710	SRP14	RNA processing	Verified by RT-PCR
F0d1a	XM_414248.2	Nucleolar RNA host gene 8	Nucleolar RNA	n.d
F0d4	XM_001234485	SMARCA5	Chromatin modification	n.d.
M5v1	NW_001471669	Estrogen receptor α	Transcription	Dorso-ventral gradient
M0d1a	ref/NC_026897.1	16S ribosomal RNA	RNA metabolism	n.d.
M6v2	XM_414131	CCDC123	Mitochondrial protein	n.d.
M17d2	XM_415003.2	Mitochondrial ribosomal protein S5	Mitochondrial protein	n.d.
M4v1	NM_001030819	ABCC4 (CFTR/MRP)	Chloride channel	n.d.
M5v4, M12v3	NW_001471710	MEKKK5	Signalling	Dorso-ventral gradient
M7v1	NW_001471526	AAT1-α	Signalling	Dorso-ventral gradient
M13v1a	XR_026877.1	Sulfatase 1 SULF 1	Signalling	n.d.
M4d2	NW_001471434	Naked cuticle (NKD1) homologue 1	Signalling	Verified by RT-PCR
F0d3	NW_001471529	Similar to EAT2	Signalling	Verified by RT-PCR
F4d1	emb/AJ720092	YWHAH	Signalling	n.d.
M5v2	XM_419938	KIAA0161, ring finger protein 144A (RNF144a)	Protein turnover	n.d.
M11d1	NW_001471685	QR1 (SPARC-like 1, Hevin)	Cell adhesion	Medio-dorsal
M5v6	NW_001471633	TEM7R/Plxdc2	Migration	Verified by RT-PCR
F0d1, F0d2	NW_001471503	Tenascin C	ECM	Verified by RT-PCR
M1v1	XM_426638	Ferric-chelate reductase 1	Iron metabolism	n.d.
M12d2	NM_001012604.	Macrophage erythroblast attacher MAEA	Migration	n.d
M1v1a	ref/XR_026897.1	Similar to MLL5	Chromatin modifications	n.d.
F4v1	XM_415718.2	RNF43	Protein turnover	n.d.

The table itemizes the BLAST accession numbers, the chick nomenclature, the function and expression of the identified clones. The code of the clone IDs is as follows: M and F followed by the number stand for the primer combination used to make cDNA, v = ventral, d = dorsal. Abbreviations used: ECM = extracellular matrix, n.d. = not determined.

**Table 2:** Sequence and chromosomal location of clones with no match to annotated chicken genes.

**Clone**	**Chromosomal location**
M2v1	no EST, on chromosome 5
TTTTTTTTTTTTGCTAAATTTCAGTGAGAAAGTCTTACTTTAGGATTCTCGAGTGTTTTCCTTCACAAAGTAGTTA AAAACCACACCCAAAAACAAACAAACAAACAAAAACACCCATAACCTATCTCCAAATTAAGTGCTACACAATAACC CCAAAGAAACAAAGTTAACACAGCCACCAGAGACATTACAACTGAGTCTTGCTAGTCTTAG	
M12d1	no EST; on chromosome 10
GCGCAAGCTTTTTTTTTTTTGCTGATAAAATGAGGGAGCATTGGGCAATCGGCAAAATAGATGGAATACCTTCTCT GCAAACAGATTTCCCAGCCCCCTCCAGGGAGATTATACAGTGGCCTTGACAAGGTAATACAACTGAAACATTTCCA TTTCTTACCCGAACGAGCCACAGTCATTTATGACTTTTCTTAAACCTCGTTGTAAT	
M12v1	no EST; on chromosome 4
TGACTCATGCATACCTGGAGACTGCCATGAGTACAGTGGCATCACTGCCGTAACACTGGAGGTACCACCTCCACAA AAGACACAGTAATTAGCAGAGAAATGGCTATTGGTGCAGCTGTAAAGTAGACAAGCACTCTTTTGGATTTCAGTTC ATTGCCTCGTTGTAAT	
M16d1	no EST; on chromosome 1
TTTTTTTTTTGCAATTGGTGTGTGAATTTTTAACTTGAGAGATGCATTGGTCTTTGTCGTGAAAAGTGCAACACAA TCACATATAATCTCTTTCCCTTTACCATA	
M17d1	no EST; on chromosome 1
TTTTTTTTTTGCAATTGGTGTGTGAATTTTTAACTTGAGAGATGCATTGGTCTTTGTCGTGAAAAGTGCAACACAA CACATATAATCTCTTTCCCTTTACCATA	
F0v2a	no EST; on chromosome 1
CTCCAAGAGTGGCAGCTAAGTGGGCTGATGGATGCTCTTCATTATTTCACTGTGTAGCTCAACTAGCTGACTGTGT AGTGATGATCTCTGAACCACTTTTTGCTGTAATTTCTGTCATGCAAAAAAAAAA	
F1v1a	no EST; on chromosome 1
TTTTTTTTTTTTGAGTTGAGAGTGTCCCTTTAAGGATTTACGGTATGGCACAGCCCAGGGTTTGCTTGGTATGGCT TTTCCTAAACTATTATTTTCTTTTGCTGGGAGACTCCGCAACTGAAAGTGCAGCTTTTTTCTTCTTCGCCGCTGTT TGCTCCCATGCTAGCTA	
F9v1	identical with EST's CN235808, BU294558, CO767427; no known homology; on chromosome 15
TTTTTTTTTTGAGCACTCGTTTATTTTTGAATAGAAAGCAAGGTAACTGTATTTGTAACATGACACTTGGCTAATT CAAACAAACAAAAACCCTGATCTTTTGCACCTTTAGAAGTGAATTGAAGCAACCCCAATCGGATTCAGTGTCTCCA CAAGTTCCATTCTTCACAACTGTGAGTGATCAGTCGCTACATAAACTGCTACTTTTAAAAAATAGCTGAATTAAAT ATATTGTCTGCTAGCTA	

The retrieved DNA sequences of the clones are given together with the location of these sequences on the chromosomes.

### Clone M11d1 – the chick QR1/Sparc-like 1

**Figure 4:**
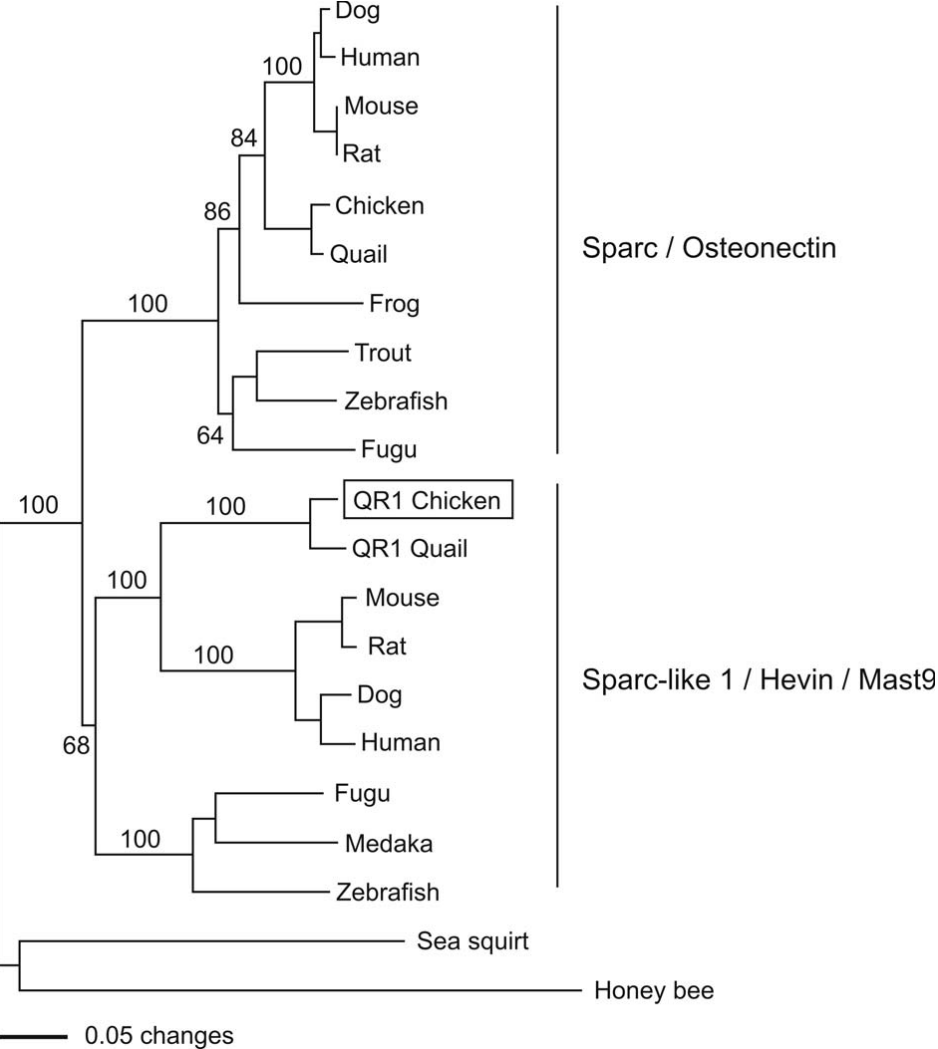
**A phylogenetic tree showing the relationships of the QR1-like genes**. Multiple sequence alignments were generated using PileUp from the GCG Wisconsin package (Version 10.3, Accelrys Inc., San Diego, CA) and ClustalX [66]. Phylogenetic analyses were performed on an alignment of 250 amino-acids using the neighbour-joining method [67] with 1,000 pseudosamples, as implemented in PAUP* [68].

**Figure 5:**
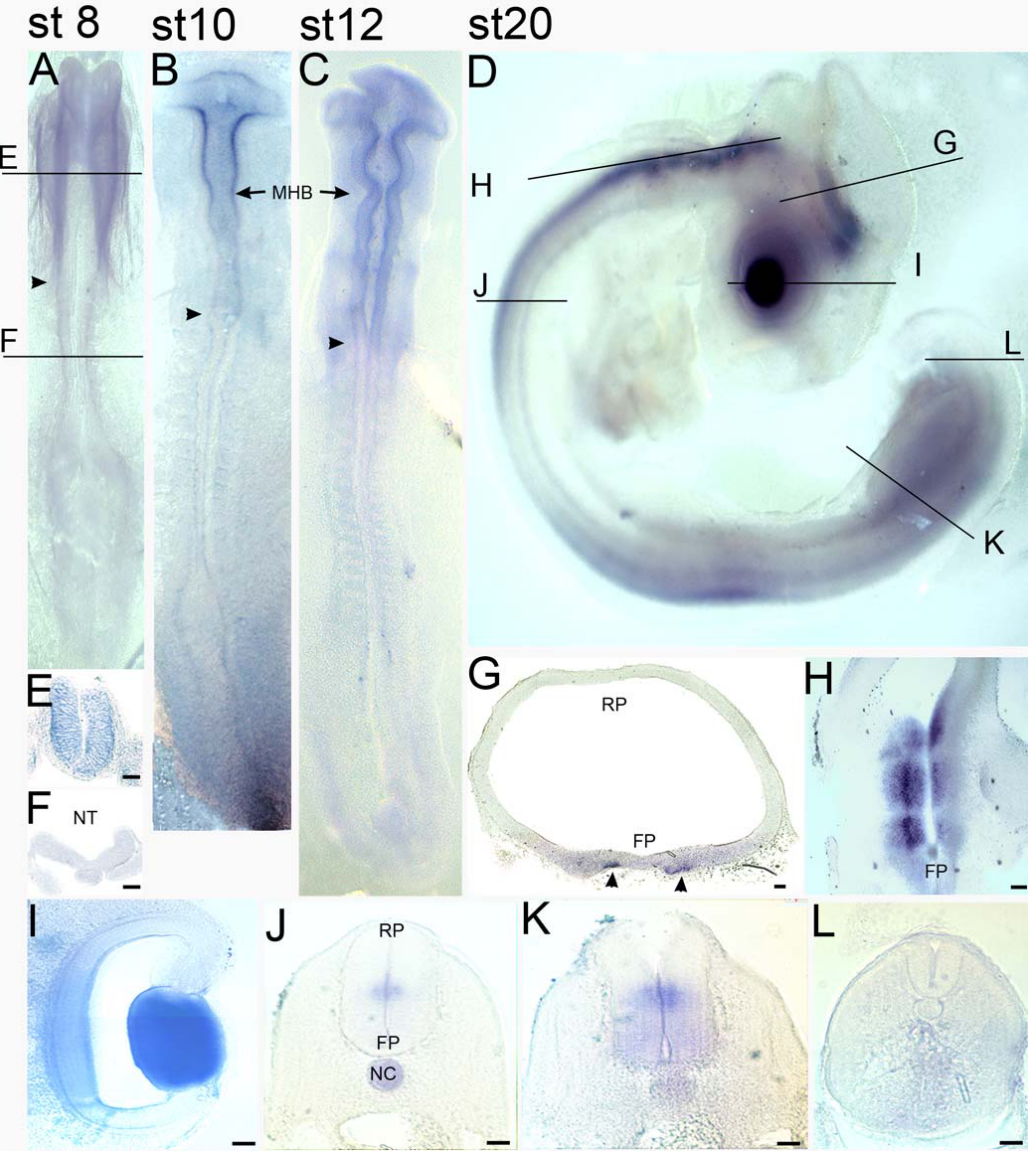
**Developmental expression profile of *QR1 *in the chick embryo**. (A-D) show RNA *in situ *hybridisation against *QR1 *of whole embryos at different developmental stages. (E-L) are coronal sections except for (H), a horizontal section. The plane of the sections is indicated in (A) and (D). (A) At HH 8 *QR1 *mRNA is detected in the entire brain region up to the boundary with the spinal cord (arrowheads). Coronal sections through HH8 show a diffuse expression of QR1 in the lateral neural tube of the brain (E) but no expression in the spinal cord (F). This expression pattern is also observed at HH 10 (B) and HH 12 (B). However, at stage 10 the expression is more intense medio-laterally (see fig. 3C). (D-I) At HH 20, *QR1 *mRNA is also present in spinal cord, lens and retina but no longer in the more anterior regions of the brain. A coronal section through the midbrain (G) at HH 20 shows *QR1 *expression in the ventral part. An horizontal section through the hindbrain (H) reveals *QR1 *expression also in ventral regions adjacent to the floor plate. Coronal sections through the spinal cord at HH stage 20 show a medial expression (J, K) and at hind limb level (K) an additional diffuse ventral expression. In the tail region of the spinal cord (L) *QR1 *expression is not detected. Scale bars are 50 μm.

We next concentrated on the identification of the extensions of the original 3' clones in order to obtain the full length coding sequences of these genes. To this end we utilised a SMART-RACE kit from Clontech to amplify extended cDNA products using the total RNA isolated from HH10 chick midbrain. We were able to obtain the 5' extension of M11d1 cDNA. BLAST search revealed this to be a known gene, *QR1*, which was originally isolated from the neural retina of quails [25,26]. Our phylogenetic analysis confirmed that this gene is orthologous to the vertebrate *SPARC-like1/Hevin/Mast9 *gene and related to but different from *SPARC/osteonectin/BM40 *(Fig. 4) [27-33]. QR1 encodes a protein, which is found in two forms, either associated with the extracellular matrix or diffusible in the medium [26]. The presence of QR1 protein in the Müller retinal glial cells coincides with cell cycle arrest and differentiation in these cells [25,26]. More recently, a screen for genes specifically expressed in the gliogenic ventral neuroepithelium of the chick at E6 identified *QR1 *expression in the medial spinal cord [34]. Its role in the astroglial cell specification was postulated, but not further investigated. An intriguing role for this family of proteins in neuronal histogenesis is revealed by the role of Sparc-like 1 protein in organizing the cortical layering. Sparc-like 1 was shown to act as an anti-adhesive molecule that is expressed by radial glia and dislodges the neurons from radial glia after they have migrated to the appropriate position in the cortex [35]. Our expression analysis of *QR1/Sparc-like 1 *during early embryonic stages demonstrated that *QR1 *was first expressed in the anterior neural tube up to the first somite, which coincides with the rhombencephalon – spinal cord boundary at HH 8 to 12 (Fig 5A–C). At these stages no expression was detected in the spinal cord. Coronal sections through the brain region confirmed a diffuse expression in the brain and no expression in the spinal cord (Fig. 5E, F) at HH 8. At later stages *QR1 *expression became more prominent in the ventral and lateral neural tube (see HH 20, Fig. 5D, G, J, K) and expression in lens tissue and in the medial part of the retina was observed (Fig. 4I). Coronal sections through the spinal cord at stage HH20 showed *QR1 *expression in the chorda (Fig. 5J, K) and around the ventricular zone of the intermediate spinal cord (Fig. 5K). At the level of the hind limbs (Fig. 5K) *QR1 *expression was also seen in more ventral parts of the spinal cord. No *QR1 *mRNA was detected in the most posterior part of the spinal cord (Fig. 5L). This expression pattern was still observed at embryonic day four (see also [34]. Given that Sparc-like 1 regulates neural migration in the murine cortex [35] and might be involved in the Mueller retinal glial cell differentiation in the retina of the quail [26], our expression data support a role of QR1/Sparc-like 1 in guiding migration and thus the cytoarchitecture of neuronal arrangement in the developing chick midbrain.

### Additional Genes identified in the Screen

The genes that were identified hitherto from our screen are summarized in table 1 and a representation of the different functional subclasses is shown in Fig. 2F. Some of the genes have been shown to have predominantly dorsal or ventral distribution within the neural tube. For example, clone M5v1 encodes an estrogen receptor α, and has been detected in ventral neurons of the rodent midbrain [36] and shown to regulate the serotonergic system of rodents and primates ([36] and references therein). The fact that we isolated this transcript from the ventral part of the midbrain validates our screening strategy. The functional implications of this early expression of estrogen receptor α remain to be investigated in future work.

Another interesting cDNA isolated with two different sets of primers used in the screen, M5v4 and M12v3, encodes a mitogen-activated protein kinase kinase kinase kinase 5 (MEKKK 5/MAP4K5). This group of serine/threonine kinases are widely implicated in intracellular signalling. Interestingly, MEKKK 5 contains an additional functional domain, referred to as the citron domain. Another kinase with citron domain, the myotonic dystrophy kinase-related Cdc42-binding kinase (MRCK alpha), has been shown to regulate neurite outgrowth in PC12 cells through binding to the small GTPases cdc42 and Rac [37]. A related kinase, germinal center kinase-related enzyme (GCKR), has recently been shown to act as a positive regulator of both canonical and non-canonical Wnt signalling in B-lymphocytes. The pathway requires Rac and leads to JNK activation [38]. Furthermore, a recent report has implicated non-canonical Wnt5a signalling and downstream activation of JNK and ROCK kinases in the guidance of facial branchiomotor neuron migration during mammalian development [39]. The non-canonical Wnt pathway also known as the planar cell polarity (PCP) pathway identified in *Drosophila *is a conserved mechanism that polarizes cells along specific axes in a tissue. PCP regulates many developmental processes including convergent extension during gastrulation, neural tube closure, hair orientation in mammals and other processes (for review see [40,41]). The most prominent role of PCP is to regulate the dynamics of polarized cells and to impart directional motility to the cells ultimately contributing to tissue morphogenesis [40,41]. It will be interesting to address a possible role of the MEKKK 5 clone in the regulation of neuronal specification and/or migration during the development of the midbrain by modulating non-canonical Wnt signalling.

A potentially intriguing clone derived from our screen is M4d2, which corresponds to a Naked cuticle homologue 1 (Nkd1) gene. Nkd1 encodes a protein with a single EF hand (a calcium-binding motif) that is most similar to the Recoverin family of myristoyl switch proteins. It was proposed to link ion fluxes to the regulation of the potency, duration or distribution of Wnt signals [42]. Nkd 1 is one of the antagonists of Wnt signalling in vertebrates [43]. In *Drosophila*, Nkd 1 has been shown to target dishevelled and thus, to antagonize the canonical Wnt signalling as well as the non-canonical PCP pathway [44]. Wnt genes are widely expressed both in the dorsal and ventral midbrain [45-47]. Thus, the role of Nkd1 as a putative antagonist of Wnt signalling might be of great interest for the development of neurons in the dorsal midbrain.

Clone M5v6 encodes a transmembrane domain protein with homology to nidogen and a plexin repeat, called Plxdc2/TEM7R. Recently, its expression was analysed in the developing mouse embryo and was found in many patterning centres of the brain at early stages, e.g., in the cortical hem, midbrain-hindbrain boundary, and the midbrain floor plate [48]. Interestingly, the authors show a striking overlap of *Plxdc2 *expression with *Wnt3a, Wnt5a and Wnt8b *expression in certain areas [48]. In an intriguing study a ligand for Plxdc2/TEM7R was identified to be cortactin [49]. Cortactin is a substrate of Src kinase and F-actin-binding protein. It has been implicated in the regulation of dendritic spine morphology and synaptic plasticity [50], in IgCAM-triggered actin assembly, which is involved in growth cone motility and guidance [51] and in mediating morphogenic cell movements during zebrafish (*Danio rerio*) gastrulation as well as in the development of the CNS [52]. Consequently, Plxdc2/TEM7R is of great interest as a potential regulator of cell migration during development and might have possible implications for the final location of different midbrain neurons.

Another class of molecules, which can modulate signalling pathways, are the heparan sulphate proteoglycans (HSPGs). These are found at the cell surface and can be modified by the addition of sulphate groups at specific sugar residues along the heparin sulphate (HS) chain. The addition or removal of sulphate groups creates a structural heterogeneity within the HSPGs, which can influence their ability to bind different proteins, notably growth factors and their receptors. In our screen, we identified Sulf1 that was encoded by clone M13v1a and is an extracellular HS 6-O-endosulfatase enzyme, which is a major regulator of HS 6-O-desulfation [53]. In *Xenopus *embryos, *Xtsulf1 *was shown to play an important role in modulating cell signalling during development by enhancing the axis-inducing activity of Wnt11 and restricting BMP and FGF signalling [54]. Sulf1 has also been shown to modulate Sonic hedgehog (Shh) activity during oligodendrocyte differentiation in the spinal cord at a later stage of chick development [55]. Sulf1 is expressed just prior to the oligodendrocyte precursor specification in the ventral spinal cord and is likely to be responsible for the change in the distribution of Shh at this time point [55].

It is intriguing that the clones, which were isolated in our screen as putative positive regulators of Wnt signalling, i.e., M5v4/M12v3/MEKKK5 and M13v1a/Sulf1 homologue were enriched in the ventral part of the midbrain. This result raises the possibility that a differential regulation of Wnt activity in the chick midbrain might constitute part of the mechanism responsible for the subsequent formation of different types of neurons and cytoarchitectural arrangements found in the dorsal and ventral parts of this brain structure. Importantly, the putative antagonist of Wnt signalling, Nkd1 encoded by M4d2, was found in the dorsal part of the chick midbrain. Other known inhibitors of Wnt signalling, e.g., Dkk1 (ACW, unpublished observations) and several SFRPs [56-58] are expressed in the dorsal midbrain as well, strengthening the possibility that differential Wnt signalling within the dorsal and ventral midbrain parts accounts at least in part for differential cytoarchitecture of this part of the brain. Recent work implicating the non-canonical Wnt5a/PCP pathway and the canonical Wnt pathway in the specification and regulation of dopaminergic progenitors and in ventral midbrain morphogenesis further supports the idea that differential Wnt regulation could account for differences in cytoarchitectural organization observed in the midbrain [59-61].

Another aspect of the general regulation of morphogenetic patterning involves proteins found in the extracellular matrix (ECM). In our screen, we identified an interesting candidate from this category of proteins, clone F0d2/F0d1, encoding *Tenascin-C*, a gene that is known to be involved in the regulation of cell migration. Tenascins are a family of large multimeric proteins of the extracellular matrix (ECM). These proteins promote weak cell adhesion and do not activate cell spreading [62]. Abnormalities have been found in the nervous system of Tenascin-C defective mice ([62] and references therein). ECM proteins are of paramount importance in the regulation of cellular migration during development. Cells with different histories move together and influence each other during migration resulting in specific cytodifferentiation events. These induction events result in early embryonic morphogenetic patterning. Thus, the interactions between groups of cells and their collective movements are in part responsible for the morphogenetic patterning during development. The regulated expression of ECM proteins can create specific microenvironments, within which groups of cells can move and thus influence the morphogenetic events. Tenascin-C, also known as cytotactin has been shown to have a dynamic and restricted pattern of expression in the chick embryo and specifically within the CNS [63]. This raises the possibility that Tenascin-C may help to guide morphogenetic movements and subsequent pattern formation by providing a temporally and spatially regulated extracellular environment for interacting groups of cells. Such regulation of cellular migration in groups could then account for the different neuronal cytoarchitecture within the dorsal and ventral parts of the chick midbrain.

The other genes identified in our screen are likely to be important in the regulation of different processes, which ultimately lead to the formation of a fully patterned midbrain in the developing chick. Both of the chromatin modifiers we found, M1v1a that corresponds to MLL5 and F0d4, which represents the SMARCA5 gene, are highly interesting as the role of chromatin modifications in the regulation of differentiation programmes of the developing organisms is just now beginning to be elucidated. It will be exciting to investigate the involvement of these genes in the regulation of patterning.

In this work we present the results of a screen performed with the final goal of identifying genes that are not only involved in the specification of neurons but also in establishing the different cytoarchitectural patterns found in the dorsal and ventral midbrain of the chick. These two regions show a distinctly different arrangement of neurons, that is a laminar organization in the dorsal/tectal part and a nuclear organization in the ventral/tegmental part. We identified several differentially expressed mRNAs from midbrains of chicks at HH9–11. The expression of one of the clones, *QR1*, has been previously shown to coincide with the cell cycle arrest in the Mueller retinal glial cells of the quail. QR1 has also been suggested to play a role in the differentiation of the astroglia in the spinal cord based on the timing and spatial distribution of its expression in the spinal cord [26,34]. More recent work implicated the mouse orthologue of QR1, Sparc-like 1 protein in restricting migration of cortical neurons [35]. We could show a very early onset of expression of *QR1 *in the chick embryo in the midbrain at HH8–10. It is interesting to postulate a possible role for QR1 in regulating some aspects of neural migration in the chick midbrain as was demonstrated for Sparc-like 1 [35]. However, proof of this will need a detailed analysis of its function *in vivo*.

Further work on the elucidation of the function of the clones obtained from our screen should provide valuable insight into the mechanisms that govern the organisation of higher neural structures, which are necessary for the correct processing of information by the animal.

## Conclusion

Here, we present evidence for the differential expression of several genes in the dorsal and ventral parts of the chick midbrain, which expands our knowledge of the molecular repertoire used during early development in the midbrain. The identified genes should also provide a useful starting platform to address questions that concern regulating mechanisms involved in the sculpturing of the different neuronal cytoarchitectural arrangement in this part of the brain.

## Methods

Fertile hen's eggs were incubated at 37°C in a humidified chamber to the required stage. The embryos were isolated and staged according to Hamburger and Hamilton [24]. Midbrains were isolated and separated into dorsal and ventral parts. These were used to prepare the cDNA libraries used in the screen.

### RNA Isolation

Total RNA from ventral and dorsal parts of the midbrains was prepared using RNeasy Kit (Qiagen) according to the manufacturer's instructions. The integrity of RNA was assessed on a 1% formaldehyde-agarose gel and the 28S and 18S rRNA were visualized using ethidium bromide. The concentration of RNA was determined by densitometry and normalization to the known amounts of standards, which were loaded onto the gels in parallel with the samples.

### Differential Display Analysis

We employed a previously published method of DD-PCR [22] using the primers previously described [23]. Briefly, combinations of 11 different oligo-dT primers (A-M) and 20 different 5' random primers (0–19) were used for the cDNA synthesis and DD-PCR (for primer sequences, see [23]. As it was previously reported [23] and personally communicated to us (Leimeister C and see [64]), that the primers M and F give the most results, we present here our initial screen with the combination of these two oligo-dT primers with the 20 5' random primers. Total RNA (300 ng) from ventral or dorsal midbrain was mixed with 2.5 μl of 20 μM oligo dT (3') primer in a total volume of 13 μl. This mixture was then heated to 75°C for 10 minutes and cooled on ice. The remainder of the reverse transcription (RT) reaction containing 5 μl 5× synthesis buffer, 2.5 μl 0.1 MDTT, 2.5 μl 250 μM dNTP-Mix, 1.5 μl H_2_O, and 50 U SuperScriptII reverse transcriptase (Gibco) were then added to the reactions containing ventral or dorsal RNA and the reactions were allowed to proceed at 37°C for 70 minutes. The enzyme was then denatured at 95°C for 10 minutes prior to further processing of the samples. The DD-PCR reactions were prepared as follows: 1 μl of the above cDNA already containing the 3'primer, 1 μl 10 × PCR buffer (100 mM Tris-HCl, pH9.0, 500 mM KCl, 15 mM MgCl_2_, 1% Triton-X 100, 2 mg/ml bovine serum albumin), 0.375 μl 100 μM dNTP, 0.625 μl 20 μM 5'random primer, 1 μCi α^32^P-dCTP, 0.5 U Taq DNA polymerase (Gibco), and 0.025 U Pwo polymerase (AGS) in a total volume of 10 μl. The reactions were overlaid with Paraffin oil and PCR carried out as follows: 60 second denaturation at 94°C, eight cycles of 45 second denaturation at 94°C, 60-second annealing at 41°C, and 120 second elongation at 72°C, followed by 15 cycles of 45 seconds at 94°C, 45 seconds at 60°C and 120 seconds at 72°C, completed by a final elongation of 5 minutes at 72°C. Subsequently, the PCR reactions were mixed with 4.5 μl denaturing sequencing dye (10 mM NaOH, 95% formamide, 0.05% bromophenol blue, 0.05% xylene cyanol) and 7 μl aliquots were separated on standard 6% polyacrylamide, 7 M urea sequencing gels. Gels were then dried and exposed to X-ray films overnight at room temperature.

### Re-amplification and Sequencing of PCR Products

Candidate bands for differentially expressed mRNAs were excised from the dried gels. For re-amplification, we used one third of the excised gel slice directly. The PCR mixture (50 μl) contained 60 pmol of upstream and downstream primers, 50 μM dNTP, and 5 U Taq polymerase. The cycling was performed as follows: 2 minutes at 94°C initially and 30 cycles at 94°C for 45 seconds, 60°C for 60 seconds annealing, and 72°C for 120 seconds elongation followed by a final extension for 5 minutes at 72°C. Amplified products were prepared for restriction digestion with Hind III (the site was incorporated into the primers facilitating the subcloning) by purification of the amplified DNA bands from the agarose gels with the Qiagen mini-columns. The bands were subcloned into pBS plasmid digested with Hind III and treated with calf alkaline phosphatase. Subcloned fragments were then used for making probes for *in situ *hybridisation and sequencing of the inserts.

### 5' Race to obtain the Extension of the original PCR Products

Race-SMART kit from Clontech was used to obtain extensions of original PCR clones isolated in the differential display-PCR (DD-PCR) screen and followed according to the manufacturer's instructions. The sequences were compared to GeneBank and EMBL databases using the BLAST program (GCG program package, Genetics Computer Group Inc., Madison, WI).

### mRNA *In Situ *Hybridisation

Differential expression of the candidate genes was verified by performing whole mount mRNA *in situ *hybridisation using the subcloned inserts at different stages of chick development. Digoxygenin-labelled RNA probes were synthesized from the cDNA clones with T3 and T7 RNA polymerases and both were used for subsequent *in situ *hybridisation analysis, whereby one copy represented sense and the other – anti-sense orientation of the corresponding transcript. The hybridisations were performed as described [65]. Embryos were photographed and subsequently embedded in gelatine-albumin mixture and sectioned on a vibratome. Sections were then mounted under coverslips using a mixture of PBS: Glycerol (1:10) and the staining visualized under a light microscope.

### Reverse Transcription and PCR amplification of identified genes

Midbrains were collected from staged chick embryos, as described above and total RNA was isolated using a Qiagen RNeasy kit according to manufacturer's instructions. We used 300 ng of total RNA from a collection of HH10 and HH16 midbrains for reverse transcription. The RNA was treated with RNase-free DNase to remove genomic DNA. M-MLV reverse transcriptase was used to synthesise the first strand of cDNA using random hexamers as primers. The cDNA template was then used directly in a PCR reaction with gene-specific primers to detect the presence of transcripts in dorsal and ventral parts of the midbrains. The primers used were as follows: GAPDH fwd 5'-ACG CCA TCA CTA TCT TCC AG-3', rev 5'-GTT GAC ACC CAT CAC AAA CA-3'; M4d2, naked cuticle homologue fwd 5'-AAC GGA AGA GTC ACA CGT GAG-3', rev 5'-GCA TGG AAG GTT TCA GGT TCA-3'; M5v6, plexin domain-containing protein 2 precursor fwd 5'-GTG GAG CTG CAG ATG TCA AAG-3', rev 5'-GCA GGA TGT CCA GAT CCT CTT-3'; F1d1, signal recognition particle 14 kD homologue fwd 5'-CTG ACA GAG CTG ACG AGA CTC-3', rev 5'-GTG ATG GTA GGG CAC TGT CC-3'; F0d2, tenascin C fwd 5'-AAG TGC TAC CGA GGT TCA GTC-3', rev 5'-CAT CTC CAA CGC TGA ACT TGT-3'; F0d3, similar to SH2 domain containing molecule EAT2 fwd 5'-GCT GGA GCC TTG TGT CTG TG-3', rev 5'-GTG CTG GAT CTA CAT GGG CAA-3'. All primers were chosen to be intron-spanning to minimize any possible contamination with genomic DNA.

### Phylogenetic analysis

Multiple sequence alignments were generated using PileUp from the GCG Wisconsin package (Version 10.3, Accelrys Inc., San Diego, CA) and ClustalX [66]. Phylogenetic analyses were performed on an alignment of 250 amino-acids using the neighbour-joining method [67] with 1,000 pseudosamples, as implemented in PAUP* [68]. QR1-related sequences were retrieved using the NCBI BLAST server . Accession numbers: SPARC-like 1: QR1 chicken *Gallus gallus *XP_420545; QR1 Japanese quail *Coturnix japonica *P23499; Human *Homo sapiens *EAX05991; Dog *Canis lupus familiaris *XP_850045; Mouse *Mus musculus *BAE22205; Rat *Rattus norvegicus *AAH61755; Zebrafish *Danio rerio *XP_695714; Fugu *Takifugu rubripes *NP_001027724; Medaka *Oryzias latipes *NP_001098371. SPARC *Homo sapiens *CAG33080; Quail *Coturnix japonica *O93390; Chicken *Gallus gallus *NP_989741; rainbow trout *Oncorhynchus mykiss *AAC99813; Dog *Canis lupus familiaris *XP_854982; Frog *Rana catesbeiana *BAD10858; Fugu *Takifugu rubripes *NP_001027722; Zebrafish *Danio rerio *AAT01213; Mouse *Mus musculus *P07214; Rat *Rattus norvegicus *P16975; Sea squirt *Ciona intestinalis *NP_001027592; Honey bee *Apis mellifera *XP_623079.

## Authors' contributions

AC executed the screen, performed some of the *in situ *hybridisations and RT-PCR and wrote the manuscript; JNV performed the phylogenetic analysis of QR1, the BLAST searches for the cDNA sequenced and edited the manuscript; AW designed, advised and supervised the project, performed some of the *in situ *hybridisations and edited the manuscript.

## References

[B1] Kiecker C, Lumsden A (2005). Compartments and their boundaries in vertebrate brain development. Nat Rev Neurosci.

[B2] Simon H, Hornbruch A, Lumsden A (1995). Independent assignment of antero-posterior and dorso-ventral positional values in the developing chick hindbrain. Current Biology.

[B3] Lumsden A, Krumlauf R (1996). Patterning the vertebrate neuroaxis. Science.

[B4] Roach FC (1945). Differentiation of the central nervous system after axial reversals of the medullary plate of *Ambystoma*. J Exp Zool.

[B5] Jacobson C-O (1964). Motor nuclei, cranial nerve roots and fibre pattern in the medulla oblongata after reversal experiments on the neural plate of axolotl larvae. I. Bilateral operations. Zool Bidrag Uppsala.

[B6] Hutchinson C (1936). Reconstitution in the nervous system following unilateral reversal of the dorsolateral axis in part of the spinal cord of *Amblystoma punctatum*. J Comp Neurol.

[B7] Huttner WB, Kosodo Y (2005). Symmetric versus asymmetric cell division during neurogenesis in the developing vertebrate central nervous system. Curr Opin Cell Biol.

[B8] Hand R, Bortone D, Mattar P, Nguyen L, Heng JI, Guerrier S, Boutt E, Peters E, Barnes AP, Parras C (2005). Phosphorylation of Neurogenin2 specifies the migration properties and the dendritic morphology of pyramidal neurons in the neocortex. Neuron.

[B9] Heng JI, Nguyen L, Castro DS, Zimmer C, Wildner H, Armant O, Skowronska-Krawczyk D, Bedogni F, Matter JM, Hevner R (2008). Neurogenin 2 controls cortical neuron migration through regulation of Rnd2. Nature.

[B10] Ge W, He F, Kim KJ, Blanchi B, Coskun V, Nguyen L, Wu X, Zhao J, Heng JI, Martinowich K (2006). Coupling of cell migration with neurogenesis by proneural bHLH factors. Proc Natl Acad Sci USA.

[B11] D'Arcangelo G, Milao GG, Chen S-C, Soares HD, Morgan JI, Curran T (1995). A protein related to extracellular matrix proteins deleted in the mouse mutant reeler. Nature.

[B12] Baba K, Sakakibara S, Setsu T, Terashima T (2007). The superficial layers of the superior colliculus are cytoarchitectually and myeloarchitectually disorganized in the reelin-deficient mouse, reeler. Brain Res.

[B13] Hirotsune S, Fleck MW, Gambello MJ, Bix GJ, Chen A, Clark GD, Ledbetter DH, McBain CJ, Wynshaw-Boris A (1998). Graded reduction of Pafah1b1 (Lis1) activity results in neuronal migration defects and early embryonic lethality. Nat Genet.

[B14] Cahana A, Escamez T, Nowakowski RS, Hayes NL, Giacobini M, von Holst A, Shmueli O, Sapir T, McConnell SK, Wurst W (2001). Targeted mutagenesis of Lis1 disrupts cortical development and LIS1 homodimerization. Proc Natl Acad Sci USA.

[B15] Tsai JW, Chen Y, Kriegstein AR, Vallee RB (2005). LIS1 RNA interference blocks neural stem cell division, morphogenesis, and motility at multiple stages. J Cell Biol.

[B16] Shu T, Ayala R, MD Nguyen, Xie Z, Gleeson JG, Tsai LH (2004). Ndel1 operates in a common pathway with LIS1 and cytoplasmic dynein to regulate cortical neuronal positioning. Neuron.

[B17] Gambello MJ, Darling DL, Yingling J, Tanaka T, Gleeson JG, Wynshaw-Boris A (2003). Multiple dose-dependent effects of Lis1 on cerebral cortical development. J Neurosci.

[B18] Feng Y, Walsh CA (2004). Mitotic spindle regulation by Nde1 controls cerebral cortical size. Neuron.

[B19] Kawauchi T, Hoshino M (2008). Molecular pathways regulating cytoskeletal organization and morphological changes in migrating neurons. Dev Neurosci.

[B20] Marin O, Valdeolmillos M, Moya F (2006). Neurons in motion: same principles for different shapes?. Trends Neurosci.

[B21] Vaage S (1973). The histogenesis of the isthmic nuclei in chick embryos (Gallus domesticus). I. A morphological study. Z Anat Entwicklungsgesch.

[B22] Liang P, Pardee AB (1992). Differential display of eukaryotic messenger RNA by means of the polymerase chain reaction. Science.

[B23] Linskens MHK, Feng J, Andrews WH, Enlow BE, Saati SM, Tonkin LA, Funk WD, Villeponteau B (1995). Cataloging altered gene expression in young and senescent cells using enhanced differential display. Nucleic Acids Research.

[B24] Hamburger V, Hamilton HL (1951). A series of of normal stages in the development of the chick embryo. Journal of Morphophology.

[B25] Guermah M, Crisanti P, Laugier D, Dezelee P, Bidou L, Pessac B, Calothy G (1991). Transcription of a quail gene expressed in embryonic retinal cells is shut off sharply at hatching. Proc Natl Acad Sci USA.

[B26] Casado FJ, Pouponnot C, Jeanny JC, Lecoq O, Calothy G, Pierani A (1996). QRI, a retina-specific gene, encodes an extracellular matrix protein exclusively expressed during neural retina differentiation. Mech Dev.

[B27] Bolander ME, Young MF, Fisher LW, Yamada Y, Termine JD (1988). Osteonectin cDNA sequence reveals potential binding regions for calcium and hydroxyapatite and shows homologies with both a basement membrane protein (SPARC) and a serine proteinase inhibitor (ovomucoid). Proc Natl Acad Sci USA.

[B28] Lankat-Buttgereit B, Mann K, Deutzmann R, Timpl R, Krieg T (1988). Cloning and complete amino acid sequences of human and murine basement membrane protein BM-40 (SPARC, osteonectin). FEBS Lett.

[B29] Mason IJ, Taylor A, Williams JG, Sage H, Hogan BL (1986). Evidence from molecular cloning that SPARC, a major product of mouse embryo parietal endoderm, is related to an endothelial cell 'culture shock' glycoprotein of Mr 43,000. Embo J.

[B30] Termine JD, Kleinman HK, Whitson SW, Conn KM, McGarvey ML, Martin GR (1981). Osteonectin, a bone-specific protein linking mineral to collagen. Cell.

[B31] Johnston IG, Paladino T, Gurd JW, Brown IR (1990). Molecular cloning of SC1: a putative brain extracellular matrix glycoprotein showing partial similarity to osteonectin/BM40/SPARC. Neuron.

[B32] Brekken RA, Sage EH (2001). SPARC, a matricellular protein: at the crossroads of cell-matrix communication. Matrix Biol.

[B33] Yan Q, Sage EH (1999). SPARC, a matricellular glycoprotein with important biological functions. J Histochem Cytochem.

[B34] Braquart-Varnier C, Danesin C, Clouscard-Martinato C, Agius E, Escalas N, Benazeraf B, Ai X, Emerson C, Cochard P, Soula C (2004). A subtractive approach to characterize genes with regionalized expression in the gliogenic ventral neuroepithelium: identification of chick sulfatase 1 as a new oligodendrocyte lineage gene. Mol Cell Neurosci.

[B35] Gongidi V, Ring C, Moody M, Brekken R, Sage EH, Rakic P, Anton ES (2004). SPARC-like 1 regulates the terminal phase of radial glia-guided migration in the cerebral cortex. Neuron.

[B36] McEwen B (2002). Estrogen actions throughout the brain. Recent Prog Horm Res.

[B37] Chen XQ, Tan I, Leung T, Lim L (1999). The myotonic dystrophy kinase-related Cdc42-binding kinase is involved in the regulation of neurite outgrowth in PC12 cells. J Biol Chem.

[B38] Shi CS, Huang NN, Harrison K, Han SB, Kehrl JH (2006). The mitogen-activated protein kinase kinase kinase kinase GCKR positively regulates canonical and noncanonical Wnt signaling in B lymphocytes. Mol Cell Biol.

[B39] Vivancos V, Chen P, Spassky N, Qian D, Dabdoub A, Kelley M, Studer M, Guthrie S (2009). Wnt activity guides facial branchiomotor neuron migration, and involves the PCP pathway and JNK and ROCK kinases. Neural Dev.

[B40] Simons M, Mlodzik M (2008). Planar cell polarity signaling: from fly development to human disease. Annu Rev Genet.

[B41] Seifert JR, Mlodzik M (2007). Frizzled/PCP signalling: a conserved mechanism regulating cell polarity and directed motility. Nat Rev Genet.

[B42] Zeng W, Wharton KA, Mack JA, Wang K, Gadbaw M, Suyama K, Klein PS, Scott MP (2000). naked cuticle encodes an inducible antagonist of Wnt signalling. Nature.

[B43] Wharton KA, Zimmermann G, Rousset R, Scott MP (2001). Vertebrate proteins related to Drosophila Naked Cuticle bind Dishevelled and antagonize Wnt signaling. Dev Biol.

[B44] Rousset R, Mack JA, Wharton KA, Axelrod JD, Cadigan KM, Fish MP, Nusse R, Scott MP (2001). Naked cuticle targets dishevelled to antagonize Wnt signal transduction. Genes Dev.

[B45] Hollyday M, McMahon JA, McMahon AP (1995). Wnt expression patterns in chick embryo nervous system. Mech Dev.

[B46] Yoshioka H, Ohuchi H, Nohno T, Fujiwara A, Tanda N, Kawakami Y, Noji S (1994). Regional expression of the Cwnt-4 gene in developing chick central nervous system in relationship to the diencephalic neuromere D2 and a dorsal domain of the spinal cord. Biochem Biophys Res Commun.

[B47] Krauss S, Korzh V, Fjose A, Johansen T (1992). Expression of four zebrafish wnt-related genes during embryogenesis. Development.

[B48] Miller SF, Summerhurst K, Runker AE, Kerjan G, Friedel RH, Chedotal A, Murphy P, Mitchell KJ (2007). Expression of Plxdc2/TEM7R in the developing nervous system of the mouse. Gene Expr Patterns.

[B49] Nanda A, Buckhaults P, Seaman S, Agrawal N, Boutin P, Shankara S, Nacht M, Teicher B, Stampfl J, Singh S (2004). Identification of a binding partner for the endothelial cell surface proteins TEM7 and TEM7R. Cancer Res.

[B50] Jaworski J, Kapitein LC, Gouveia SM, Dortland BR, Wulf PS, Grigoriev I, Camera P, Spangler SA, Di Stefano P, Demmers J (2009). Dynamic microtubules regulate dendritic spine morphology and synaptic plasticity. Neuron.

[B51] Decourt B, Munnamalai V, Lee AC, Sanchez L, Suter DM (2009). Cortactin colocalizes with filopodial actin and accumulates at IgCAM adhesion sites in Aplysia growth cones. J Neurosci Res.

[B52] Yu D, Zhang P, Zhan X (2005). Cortactin mediated morphogenic cell movements during zebrafish (Danio rerio) gastrulation. Sci China C Life Sci.

[B53] Ai X, Kitazawa T, Do AT, Kusche-Gullberg M, Labosky PA, Emerson CP (2007). SULF1 and SULF2 regulate heparan sulfate-mediated GDNF signaling for esophageal innervation. Development.

[B54] Freeman SD, Moore WM, Guiral EC, Holme AD, Turnbull JE, Pownall ME (2008). Extracellular regulation of developmental cell signaling by XtSulf1. Dev Biol.

[B55] Danesin C, Agius E, Escalas N, Ai X, Emerson C, Cochard P, Soula C (2006). Ventral neural progenitors switch toward an oligodendroglial fate in response to increased Sonic hedgehog (Shh) activity: involvement of Sulfatase 1 in modulating Shh signaling in the ventral spinal cord. J Neurosci.

[B56] Terry K, Magan H, Baranski M, Burrus LW (2000). Sfrp-1 and sfrp-2 are expressed in overlapping and distinct domains during chick development. Mech Dev.

[B57] Ladher RK, Church VL, Allen S, Robson L, Abdelfattah A, Brown NA, Hattersley G, Rosen V, Luyten FP, Dale L (2000). Cloning and expression of the Wnt antagonists Sfrp-2 and Frzb during chick development. Dev Biol.

[B58] Baranski M, Berdougo E, Sandler JS, Darnell DK, Burrus LW (2000). The dynamic expression pattern of frzb-1 suggests multiple roles in chick development. Dev Biol.

[B59] Andersson ER, Prakash N, Cajanek L, Minina E, Bryja V, Bryjova L, Yamaguchi TP, Hall AC, Wurst W, Arenas E (2008). Wnt5a regulates ventral midbrain morphogenesis and the development of A9–A10 dopaminergic cells in vivo. PLoS ONE.

[B60] Joksimovic M, Yun BA, Kittappa R, Anderegg AM, Chang WW, Taketo MM, McKay RD, Awatramani RB (2009). Wnt antagonism of Shh facilitates midbrain floor plate neurogenesis. Nat Neurosci.

[B61] Prakash N, Brodski C, Naserke T, Puelles E, Gogoi R, Hall A, Panhuysen M, Echevarria D, Sussel L, Weisenhorn DM (2006). A Wnt1-regulated genetic network controls the identity and fate of midbrain-dopaminergic progenitors in vivo. Development.

[B62] Chiquet-Ehrismann R (2004). Tenascins. Int J Biochem Cell Biol.

[B63] Crossin KL, Hoffman S, Grumet M, Thiery JP, Edelman GM (1986). Site-restricted expression of cytotactin during development of the chicken embryo. J Cell Biol.

[B64] Leimeister C, Bach A, Woolf AS, Gessler M (1999). Screen for genes regulated during early kidney morphogenesis. Dev Genet.

[B65] Henrique D, Adam J, Myat A, Chitnis A, Lewis J, Ish-Horowicz D (1995). Expression of a Delta homologue in prospective neurons in the chick. Nature.

[B66] Thompson JD, Gibson TJ, Plewniak F, Jeanmougin F, Higgins DG (1997). The CLUSTAL_X windows interface: flexible strategies for multiple sequence alignment aided by quality analysis tools. Nucleic Acids Res.

[B67] Saitou N, Nei M (1987). The neighbor-joining method: a new method for reconstructing phylogenetic trees. Mol Biol Evol.

[B68] Rogers JS, Swofford DL (1998). A fast method for approximating maximum likelihoods of phylogenetic trees from nucleotide sequences. Syst Biol.

